# Effect of extracytoplasmic function sigma factors on autoaggregation, hemagglutination, and cell surface properties of *Porphyromonas gingivalis*

**DOI:** 10.1371/journal.pone.0185027

**Published:** 2017-09-20

**Authors:** Kazutaka Fujise, Yuichiro Kikuchi, Eitoyo Kokubu, Kazuko Okamoto-Shibayama, Kazuyuki Ishihara

**Affiliations:** 1 Department of Microbiology, Tokyo Dental College, Chiyoda-ku, Tokyo, Japan; 2 Oral Health Science Center, Tokyo Dental College, Chiyoda-ku, Tokyo, Japan; Centre National de la Recherche Scientifique, Aix-Marseille Université, FRANCE

## Abstract

*Porphyromonas gingivalis* is a bacterium frequently isolated from chronic periodontal lesions and is involved in the development of chronic periodontitis. To colonize the gingival crevice, *P*. *gingivalis* has to adapt to environmental stresses. Microbial gene expression is regulated by transcription factors such as those in two-component systems and extracytoplasmic function (ECF) sigma factors. ECF sigma factors are involved in the regulation of environmental stress response genes; however, the roles of individual ECF sigma factors are largely unknown. The purpose of this study was to investigate the functions, including autoaggregation, hemagglutination, gingipain activity, susceptibility to antimicrobial agents, and surface structure formation, of *P*. *gingivalis* ECF sigma factors encoded by SigP (PGN_0274), SigCH (PGN_0319), PGN_0450, PGN_0970, and SigH (PGN_1740). Various physiological aspects of the *sigP* mutant were affected; autoaggregation was significantly decreased at 60 min (*p* < 0.001), hemagglutination activity was markedly reduced, and enzymatic activities of Kgp and Rgps were significantly decreased (*p* < 0.001). The other mutants also showed approximately 50% reduction in Rgps activity. Kgp activity was significantly reduced in the *sigH* mutant (*p* < 0.001). No significant differences in susceptibilities to tetracycline and ofloxacin were observed in the mutants compared to those of the wild-type strain. However, the *sigP* mutant displayed an increased susceptibility to ampicillin, whereas the *PGN_0450* and *sigH* mutants showed reduced susceptibility. Transmission electron microscopy images revealed increased levels of outer membrane vesicles formed at the cell surfaces of the *sigP* mutant. These results indicate that SigP is important for bacterial surface-associated activities, including gingipain activity, autoaggregation, hemagglutination, vesicle formation, and antimicrobial susceptibility.

## Introduction

Bacteria are exposed to physiological stresses such as temperature, oxygen tension, and pH in natural environments that they inhabit. They adapt to these environmental stresses using multiple regulatory pathways [1, 2]. Extracytoplasmic function (ECF) affects bacterial signaling [3] and is associated with signals relaying extracytoplasmic conditions to the cytoplasm. ECF sigma factors, which are the largest group of alternative sigma factors, are activated by environmental stresses and recruit RNA polymerases to directly transcribe genes that encode proteins with environment-specific functions [4, 5].

*Porphyromonas gingivalis* is a Gram-negative anaerobic bacterium frequently isolated, together with *Treponema denticola* and *Tannerella forsythia*, from human chronic periodontal lesions [6]. This microorganism produces cell surface-associated virulence factors, such as cysteine proteases (gingipains), lipopolysaccharides, and fimbriae [7–9], and can invade epithelial cells [10]. It is considered to be a key microorganism in the development of periodontal disease [11]. To colonize the gingival crevice, *P*. *gingivalis* has to adapt to the environment. Six putative ECF sigma factors, namely, PGN_0274, PGN_0319, PGN_0450, PGN_0970, PGN_1108, and PGN_1740, were identified in the genome of *P*. *gingivalis* ATCC 33277 (GenBank: AP009380) [12].

The functions of ECF sigma factor have been reported in previous studies. For example, PG1318 in strain W83 [*P*. *gingivalis* ATCC 33277 open reading frame (ORF) number: PGN_1108] is involved in the regulation of mutation frequency in *P*. *gingivalis* [13]. *P*. *gingivalis* W83 mutants PG0162 (*P*. *gingivalis* ATCC 33277 ORF number: PGN_0274) and PG1660 (ATCC 33277 ORF number: PGN_0450) showed decreased gingipain activity [14]. PG1827 (SigH) in strain W83 (ATCC 33277 ORF number: PGN_1740) plays a key role in regulation and adaptation to oxidative stress [15]. PGN_0274 and SigH play key roles in biofilm formation [16]. The expression of approximately 24% of *P*. *gingivalis* W83 genes was shown to be altered with overexpression of PG0162 [17]. SigP (PGN_0274) regulates the type IX secretion system (T9SS) via the two-component system PorXY[18]. SigCH (PGN_0319) acts as a direct regulator of *cdhR* and *hmuYR*, and involved in hemin utilization [19]. However, the involvement of ECF sigma factors in shaping *P*. *gingivalis* cell surface characteristics is largely unknown. In the present study, we used *sigP*, *sigCH*, *PGN_0450*, *PGN_0970*, and *sigH* mutants to investigate the roles of ECF sigma factors, focusing on *P*. *gingivalis* cell surface characteristics.

## Materials and methods

### Bacterial culture conditions

Bacterial strains used in this study are listed in Table 1. *P*. *gingivalis* ATCC 33277 (wild-type) and *sigP*, *sigCH*, *PGN_0450*, *PGN_0970*, and *sigH* mutants were used. *P*. *gingivalis* strains were maintained anaerobically (10% CO_2_, 10% H_2_, and 80% N_2_) at 37°C on enriched tryptic soy (TS) agar (Becton Dickinson, Franklin Lakes, NJ) supplemented with 0.5% brain heart infusion (BHI) (Becton Dickinson), 0.1% cysteine (Wako Pure Chemical Industries, Osaka, Japan), 5 μg/mL hemin (Sigma-Aldrich, St. Louis, MO, USA), 0.5 μg/mL menadione (Nacalai Tesque, Kyoto, Japan), and 5% defibrinated horse blood (Nippon Bio-Test Laboratories, Tokyo, Japan). When required, erythromycin (15 μg/mL) was added to the medium. For liquid cultures, *P*. *gingivalis* cells were grown in enriched BHI medium supplemented with 0.5% yeast extract (Becton Dickinson), 0.1% cysteine, 5 μg/mL hemin, and 0.5 μg/mL menadione [20].

**Table 1:** Bacterial strains and plasmids used in this study.

Strain or plasmid	Description	Reference or source
***Escherichia coli* strain**		
DH5α	General-purpose host strain for cloning	Thermo Fisher Scientific
***Porphyromonas gingivalis* strain**		
ATCC 33277	wild type	American Type Culture Collection
KDP314	*sigP* (*PGN_0274*)::*ermF ermAM*, Em^r^	[16]
KDP315	*sigCH* (*PGN_0319*)::*ermF ermAM*, Em^r^	[16]
KDP316	*PGN_0450*::*ermF ermAM*, Em^r^	[16]
KDP317	*PGN_0970*::*ermF ermAM*, Em^r^	[16]
KDP319	*sigH* (*PGN_1740*)::*ermF ermAM*, Em^r^	[16]
KDP314C	*sigP*::*ermF ermAM*,Em^r^ pT-COW-*sigP*, Tc^r^	[16]
W83	wild type	[13]
KDP307	*sigP* (*PG0162*)::*ermF ermAM*, Em^r^	this study
KDP307C	*sigP* (*PG0162*)::*ermF ermAM*, *fimA*::*tetQ sigP*^*+*^ *sigP* ^+^	this study
***Escherichia coli* plasmid**		
pGEM-T Easy	Ap^r^, plasmid vector for TA cloning	Promega
pKD355	Ap^r^, contains the *ermF ermAM* DNA cassette between *Eco*RI and *Bam*HI of pUC18	[13]
pKD806	Ap^r^, contains the 2.0-kb PCR-amplified fragment (*PG0162* region) in pGEM-T Easy	this study
pKD811	Ap^r^ Em^r^, contains the *ermF ermAM* DNA cassette at *BamHI* site within PG0162 of pKD806	this study
pKD703	Ap^r^, contains 0.77 kb *fimA*-upstream and 0.78 kb *fimA* downstream DNA fragments	[21]
pKD713	Ap^r^, Tc^r^ contains the *tetQ* DNA cassette at *Bam*HI site of pKD703	[21]
pKD831	Ap^r^, Tc^r^ contains the *1*.*28* kb *sigP* (*PG0162*) region at DNA cassette at *Eco*RI site of pKD713	this study

### Mice

The animal protocols were approved by the Tokyo Dental College Animal Ethics Committee (Approval Number: 290601). All experiments were performed in accordance with the Guidelines for the Treatment of Experimental Animals at Tokyo Dental College. Female BALB/c mice (8–10 weeks of age) obtained from Sankyo Labo Service (Tokyo, Japan) were housed five per cage and allowed to acclimate for 1 week. All surgery was performed under pentobarbital (30 mg/kg of body weight) and sevoflurane anesthesia. All mice were euthanized with pentobarbital (200 mg/kg of body weight) at the end of the study. All efforts were made to minimize animal suffering and to reduce the number of mice used.

### Construction of ECF sigma factor mutants and complemented strains

ECF sigma factor mutants in ATCC 33277 were generated by double recombination of the targeted genes and the introduction of erythromycin resistance genes as previously described [16]. Flanking fragments, both upstream and downstream of each ECF sigma factor, were amplified by PCR from the chromosomal DNA of *P*. *gingivalis* ATCC 33277. The 2.1-kb *ermF*-*ermAM* cassette was inserted into the *Bam*HI or *Bgl*II site within the ECF sigma factor gene to yield plasmids for mutagenesis. The plasmids were digested with *Not*I and introduced into *P*. *gingivalis* ATCC 33277 by electroporation. For complementation of SigP, the whole *sigP* region with its upstream and downstream flanking regions (0.5 kb) was PCR amplified from the genomic DNA, and the complete fragment was cloned into the shuttle vector pT-COW [22]. The resulting plasmid was introduced into *sigP* mutant by electroporation. Transformants were selected on enriched TS agar containing 15 μg/mL erythromycin and 1 μg/mL tetracycline.

To generate a *sigP* mutant in W83, the coding region of *sigP* (PG0162) was amplified by PCR from the chromosomal DNA of *P*. *gingivalis* W83 using primers PG0162-U-F and PG0162-D-R. The amplified DNA fragment (2.0 kb) was cloned into a pGEM-T Easy vector, resulting in pKD806. The *ermF*-*ermAM* cassette of pKD355 was inserted into the *Bam*HI site within *sigP* of pKD806 to yield pKD811. pKD811 was digested with *Not*I and introduced into *P*. *gingivalis* W83 by electroporation, resulting in strain KDP307. To construct the *sigP*^+^-complemented mutant, the whole *sigP* gene region with its upstream and downstream flanking regions (1.28 kb) was PCR-amplified from W83 chromosomal DNA using the primer pair PG0162-COMP-U-F and PG0162-COMP-D-R, digested with *Bam*HI and *Pst*I, and inserted into the *Bam*HI and *Pst*I site of pKD713, resulting in pKD831. pKD831 was linearized by *Not*I digestion and introduced into KDP 307, resulting in strain KDP307C.

### Autoaggregation assay

The autoaggregation assay was performed as described previously [23]. Briefly, *P*. *gingivalis* strains were cultured anaerobically at 37°C for 2 days in enriched BHI broth and then harvested by centrifugation at 8,000 × *g* for 10 min at 4°C. The pellets were gently washed with 20 mM phosphate-buffered saline (PBS) twice and resuspended in the same buffer. Suspension turbidities were adjusted to OD_660_ = 1.0 using a spectrophotometer (Mini Photo 518R; Taitec, Saitama, Japan). Aliquots (5 mL) of each sample were placed in test tubes (18-mm diameter) and shaken at 37°C at a speed of 120 strokes/min. Autoaggregation was monitored at 25°C, by measuring the decrease in turbidity (660 nm) for 60 min.

### Hydrophobicity assay

A surface hydrophobicity assay was performed according to the method of Naito et al. [24]. Briefly, *P*. *gingivalis* strains were cultured anaerobically at 37°C for 2 days in enriched BHI broth and then harvested by centrifugation at 8,000 × *g* for 10 min at 4°C. The pellets were gently washed with the phosphate urea magnesium sulfate (PUM) buffer (pH 7.2) three times and then resuspended in the same buffer. Suspension turbidities were adjusted to OD_550_ = 0.6 using a spectrophotometer (Shimadzu UV-2550; Shimadzu, Kyoto, Japan). A sample (3 mL) of each bacterial suspension was placed in a test tube (13-mm diameter) and mixed with 400 μL of hexadecane (Wako Pure Chemical Industries). Following vigorous shaking, the tubes were allowed to stand for 15 min. The percent hydrophobicity was calculated as follows: [(A_550_ without hexadecane − A_550_ with hexadecane) / A_550_ without hexadecane)] × 100. Each isolate was assayed twice, and the values obtained were averaged. Experiments were performed with triplicate samples and were repeated three times to verify the results.

### Hemagglutination activity (HA) assay

Hemagglutination assay was performed as described previously [13]. Briefly, *P*. *gingivalis* cells were cultured in enriched BHI medium to the stationary phase, and then the samples were centrifuged, washed with 10 mM PBS (pH 7.4), and suspended in PBS to adjust the turbidity to 1.8 (OD_600_) as determined by a spectrophotometer (Shimadzu UV-2550). Bacterial suspensions were diluted twofold in PBS using a round-bottom microtiter plate (Sumitomo Bakelite Co., Ltd., Tokyo, Japan), and equal volumes of 1% horse erythrocytes suspended in PBS were added. HA was evaluated visually after 3 h of incubation at room temperature.

### Enzymatic assays

Lys-X cysteine proteinase (Kgp) and Arg-X cysteine proteinase (RgpA and RgpB) activities were determined using synthetic substrates N-(*p*-tosyl)-Gly-Pro-Lys 4-nitroanilide acetate salt (Sigma-Aldrich Japan, Tokyo, Japan) and benzoyl- l-arginine *p*-nitroanilide monohydrochloride, respectively (Peptide Institute, Inc., Osaka, Japan). Samples were prepared as previously described, with some modifications [13]. Briefly, the absorbances (OD_600_) of *P*. *gingivalis* cultures were measured after 48 h of anaerobic incubation at 37°C. Culture aliquots (3.0 mL) were centrifuged at 8,000 × *g* for 10 min at 4°C, and the resulting supernatant was used as “culture supernatant” in the assays. Pelleted cells were washed twice with 10 mM HEPES/NaOH (pH 7.4) and then suspended in the same buffer (OD_600_ = 1.0) for use as “whole cells.” Gingipain activities were determined in 200 μL of reaction buffer containing 0.2 mM substrate, 50 mM Tris-HCl (pH 8.0), and 10 mM DTT. Reactions were initiated by the addition of 4.0 μL of culture supernatant or whole cells. The reaction mixtures were incubated at 37°C for 30 min. Acetic acid (50%, 40 μL) was added, and the release of the cleaved product, *p*-nitroanilide, was determined by absorbance measurements at 405 nm using a Spectra Max M5 (Molecular Device, Sunnyvale, CA, USA). Proteinase activity values were divided by OD_600_ cell density values to normalize the data to OD_600_ units.

### Quantitative reverse-transcription PCR (qRT-PCR)

Total RNA was extracted from *P*. *gingivalis* cells grown to an OD_660_ of 0.6–0.8 using TRIzol reagent (Thermo Fisher Scientific, MA, USA) and following the protocol described by the manufacturer. The RNA samples were treated using the TURBO DNA-free Kit (Thermo Fisher Scientific, MA, USA) at 37°C for 15 min to eliminate contaminating genomic DNA, and then PCR was performed to confirm the lack of detectable genomic DNA in the RNA samples. RNA was reverse transcribed using the ReverTra Ace qPCR RT Master Mix with gDNA Remover (Toyobo, Osaka, Japan) following the manufacturer’s instructions. Each reaction mixture contained 1 μL of cDNA mixed with 10 μL of Taqman Fast Universal PCR MasterMix (Thermo Fisher Scientific), 1 μL gene-specific primers and probes mixture (Table 2) and 8 **μL** of RNase-free water. PCR was performed using a StepOne Plus Real-Time PCR System (Thermo Fisher Scientific). Experiments were performed in triplicate with three biologically independent replicates. Data were normalized to the mRNA level of the housekeeping gene *glk* [25]. All primers used in this study are listed in Table 2.

**Table 2:** Primers and probes used in this study.

Primer/probe	Nucleotide Sequence (5′-3′)
For construction of the *sigP* (*PG0162*) mutant and complemented strain	
PG0162-U-F	TCGACAGTTGATTGCCGAT
PG0162-D-R	TGATTCGTTCCCCAAGGTTT
PG0162-COMP-U-F	CCTGCAGGCTGCTACTGTCTCGGACGTG
PG0162-COMP-D-R	CCCTAGGGCAAACACAAACTCCGACGT
For qRT-PCR	
PGN_0381 (*glk*) probe	56-FAM/TTCGAGACT/ZEN/ACGGGAGCCATCCTC/3IABkFQ
PGN_0381 (*glk*)-F	GATGGCCTTGCTCAAGAGATA
PGN_0381 (*glk*)-R	GGTCACCTGCTTTGGTAAGA
PGN_1970 (*rgpA*) probe	56-FAM/ATGCAACCA/ZEN/CTAATACCGCTCGCA/3IABkFQ
PGN_1970 (*rgpA*)-F	AGTAACGCTCAAGTGGGATG
PGN_1970 (*rgpA*)-R	CATCGCTGACTGACAGAAGAA
PGN_1466 (*rgpB*) probe	56-FAM/AACTGTAGA/ZEN/AAGTCCTGCTGCCGG/3IABkFQ
PGN_1466 (*rgpB*)-F	GCCGACGTAGCCAATGATAA
PGN_1466 (*rgpB*)-R	CGGCCGTTCATATCGAAGAT
PGN_1728 (*kgp*) probe	56-FAM/TGGCGATGG/ZEN/TTCGGTTATGCCTTA/3IABkFQ
PGN_1728 (*kgp*) -F	CCGCTACTCATGCTGGAAATA
PGN_1728 (*kgp*) -R	CAGAGAAGCAGGAAGCGTATAA
PGN_1733 (*hagA*) probe	56-FAM/AAGACTGCT/ZEN/CCTTCTGTGACGCAC/3IABkFQ
PGN_1728 (*hagA*) -F	CGCCGTCCTATTATCCCTATTG
PGN_1728 (*hagA*) -R	CGGAGATCCTTAACCTTGGATG

### Antimicrobial susceptibility testing

Susceptibility of *P*. *gingivalis* strains to three antimicrobial agents, ampicillin, tetracycline, and ofloxacin, was investigated as described previously [26]. Briefly, TS blood agar plates with doubling concentrations of 0.125–2.0 μg/mL of ampicillin, 0.0625–0.5 μg/mL of tetracycline, and 0.0625–0.5 μg/mL of ofloxacin were made. The *P*. *gingivalis* strains were cultured overnight in enriched BHI broth. The turbidity of late log phase cultures (OD_660_) was adjusted to 0.2 with fresh medium, and the diluted cultures were grown overnight. The new late log phase cultures were diluted in the same manner to OD_660_ = 0.2. Culture aliquots (2 μL) were spotted on TS blood agar plates with or without antimicrobials and incubated anaerobically at 37°C for 7 days. Minimum inhibitory concentration (MIC) values were considered the lowest antimicrobial agent concentrations that resulted in no growth of the bacteria in the inoculum. Each antibiotic sensitivity assay was performed at least three times.

### Transmission electron microscopy (TEM)

*P*. *gingivalis* strains were cultured in enriched BHI broth at 37°C for 2 days. Late exponential phase cultures (OD_660_ = 0.8) were harvested and then fixed with 2.5% glutaraldehyde for 2 h at 4°C. Bacterial cells were washed with PBS, fixed with 1% aqueous OsO_4_ for 2 h, and washed again with PBS. The cells were dehydrated by incubation in an ethanol series (70–100% for 15 min) and suspended in propylene oxide solution for 30 min. Samples were embedded in an Epon 812/resin mixture and allowed to polymerize at 60°C for 48 h. Thin sections (70 nm) were cut by cryo-ultramicrotome (Leica Biosystems, Wetzlar, Germany), stained for less than 1 min with 4% uranyl acetate and 0.2% lead citrate solutions, and examined under TEM (H-7650, Hitachi High-Technologies Corporation, Tokyo, Japan). The outer membrane vesicles (OMVs) were semi-quantified by manually counting the number of OMVs per 1.4 μm^2^ in 15 specimens (one cell per field) [27, 28]. In addition, inter-membrane distances (between the outer and cytoplasmic membranes) of at least 20 specimens were determined from TEM images using ImageJ software (version 1.44p; http://imagej.nih.gov/ij/).

### Mouse virulence assay

The virulence of the *P*. *gingivalis* W83 *sigP* mutant and the complemented *sigP* mutant strain were determined by mouse subcutaneous infection experiments [29, 30]. Each *P*. *gingivalis* strain was cultured 2 days in enriched BHI broth until an OD_600_ = 1.0 was reached. The concentration of this sample was approximately 1×10^9^ CFU/mL. The samples were centrifuged and suspended in fresh BHI to adjust the concentration of approximately 1 × 10^11^ CFU/mL. For each *P*. *gingivalis* strain, five mice were inoculated subcutaneously with 0.1 mL of bacterial suspension at two sites on the depilated dorsal surface (0.2 mL per mouse). Survival was observed daily. Three sets of experiments were carried out, using 15 mice in total. For data analysis, Kaplan-Meier plots were constructed, and the log rank test was used to evaluate the differences in mean survival rates between mice infected with the W83 parent strain, *sigP* mutant, and complemented strain in three experiments using GraphPad Prism (version 7.0 for Windows, GraphPad Software, La Jolla, CA, USA).

### Statistical analysis

One-way ANOVA test and Dunnett's or Kruskal-Wallis multiple comparison test were used to compare the data for *P*. *gingivalis* wild type and the ECF sigma factor mutants using the GraphPad Prism, except for data from the mouse virulence assay. Differences were considered significant if *p* < 0.05.

## Results

### Autoaggregation is most affected in *P*. *gingivalis sigP* mutant

**Fig 1:**
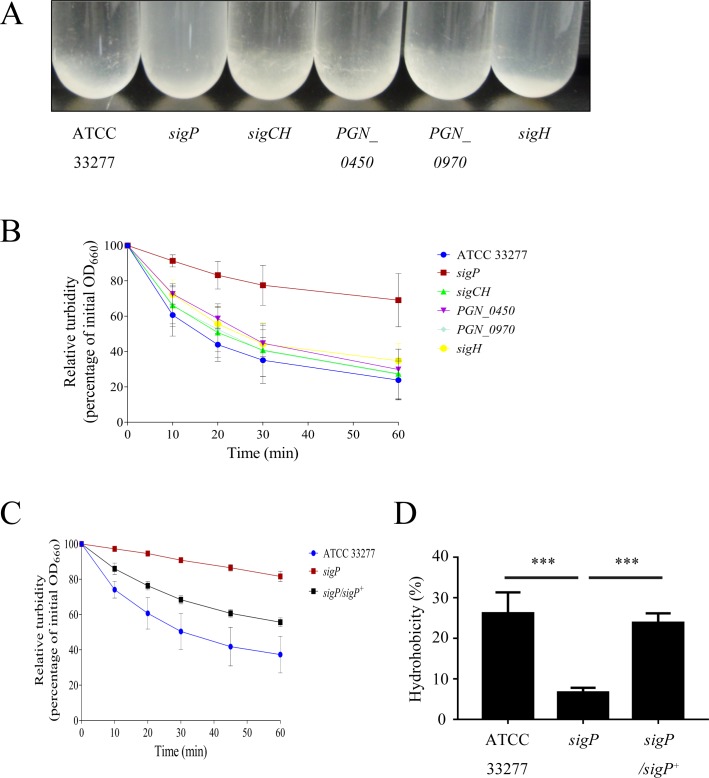
Autoaggregation of *Porphyromonas gingivalis* wild type and extracytoplasmic function (ECF) sigma factor mutants. (A) Representative images of strains at 60 min. The OD_660_ of ECF sigma factor mutants (B) or the *sigP* mutant and *sigP*-complemented strain (C) were measured at 0, 10, 20, 30, and 60 min. Relative turbidities were calculated as follows: relative turbidity (%) = (turbidity at each time point) / (turbidity at 0 min) × 100. (D) Hydrophobicity. All assays were performed three times, and means ± SD (standard deviations) are shown.

Several studies have indicated that *P*. *gingivalis* autoaggregation is associated with cell wall components and characteristics such as fimbriae, Kgp, and hydrophobicity [23, 31, 32]. We compared the autoaggregation of wild-type cells and ECF sigma factor mutants to determine whether ECF sigma factors modulate cell wall architecture. As shown in Fig 1A and 1B, autoaggregation of the *sigP* mutant was significantly reduced at all time points (10–60 min) compared to that of the wild type and *sigCH* and *PGN_0970* mutants (*p* < 0.05). The *sigP* mutant also showed a significant reduction in autoaggregation when compared with *PGN_0450* and *sigH* mutants at all time points except at 20 and 30 min (*p* < 0.05). To determine whether the reduction in autoaggregation was caused by the deletion of SigP, we constructed the SigP-complemented strain. Although a significant difference compared to the *sigP* mutant, the SigP-complemented strain failed to restore the wild-type phenotype (Fig 1C). In addition, the hydrophobicity assay revealed that the *sigP* mutant was less hydrophobic than the wild-type and complemented strains (Fig 1D).

### *P*. *gingivalis sigP* and *sigH* mutants display reduced HA

**Fig 2:**
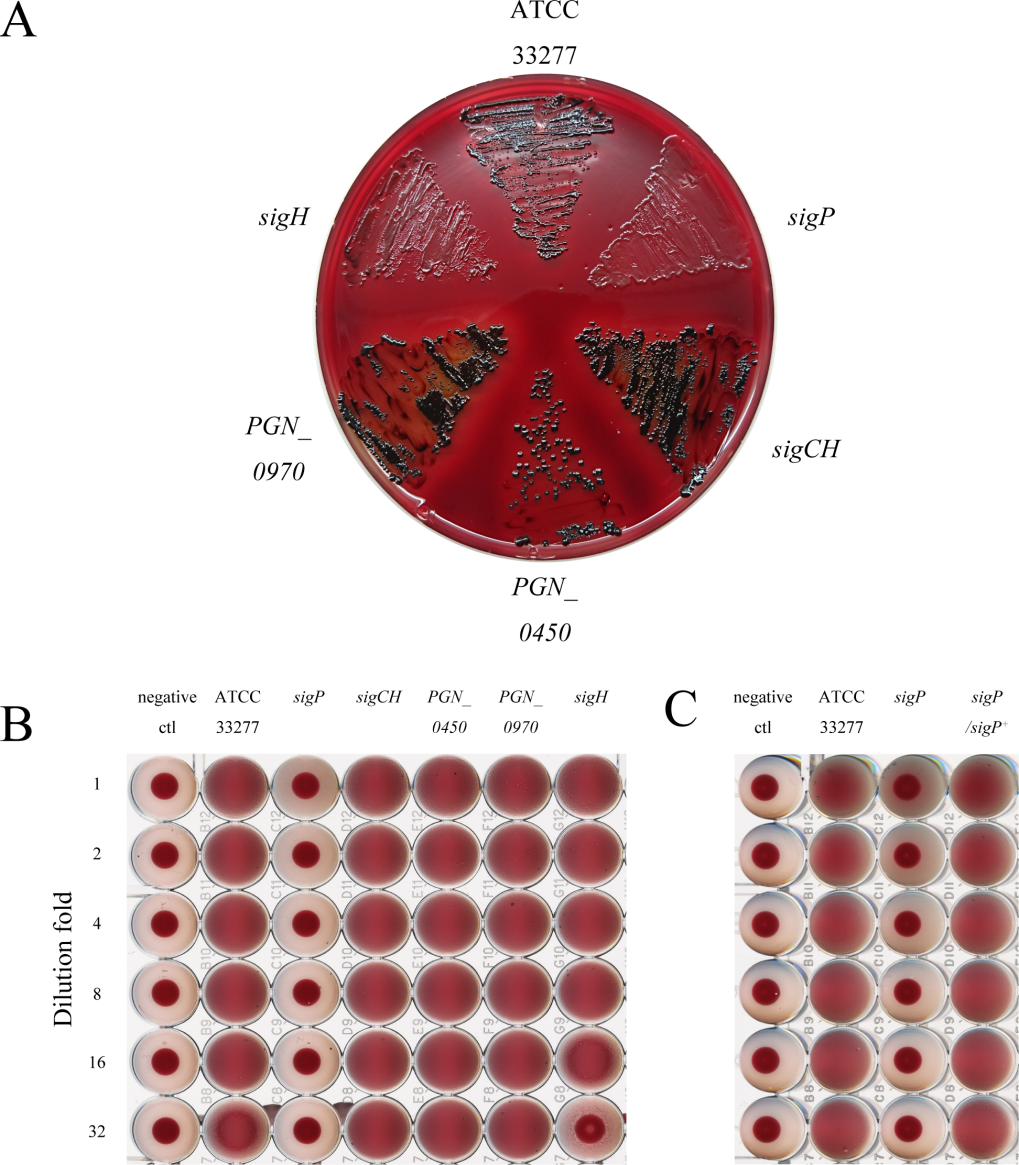
Morphology and hemagglutinin activity of *Porphyromonas gingivalis* wild type and ECF sigma factor mutant strains. (A) Colony pigmentation. The strains were grown anaerobically on enriched tryptic soy agar plates supplemented with 5% defibrinated horse blood at 37°C for 7 days. (B and C) Hemagglutination. The OD_600_ of *P*. *gingivalis* ECF sigma factor mutant (B) or the *sigP* mutant and *sigP*-complemented strain (C) cultures were adjusted to 1.8, and the cultures were diluted 1 to 32 times. After diluting, horse erythrocyte suspensions (1% in PBS) were added to each of the wells.

To further confirm the relation between ECF sigma factors and colonial pigmentation, we examined black pigmentation in colonies. As shown in Fig 2A, the *sigP* mutant and *sigH* mutants exhibited non-pigmented or less-pigmented colonies, whereas the wild type, *sigCH*, *PGN_0450*, and *PGN_0970* mutants showed black pigmentation. Next, we compared the HAs of ECF sigma factor mutants, because membrane-associated protein Hgp44 is important for hemagglutination in *P*. *gingivalis* [33]. The results are shown in Fig 2B and 2C. The *sigP* mutant exhibited hemagglutination titers more than five dilutions higher than those of the wild type. The *sigH* mutant exhibited hemagglutination titers one dilution higher than those of the wild type. The *sigP*-complemented strain restored the HA levels to the same as those of the wild type (Fig 2C).

### Rgp and Kgp activities are differentially affected in *P*. *gingivalis* ECF sigma factor mutants

**Fig 3:**
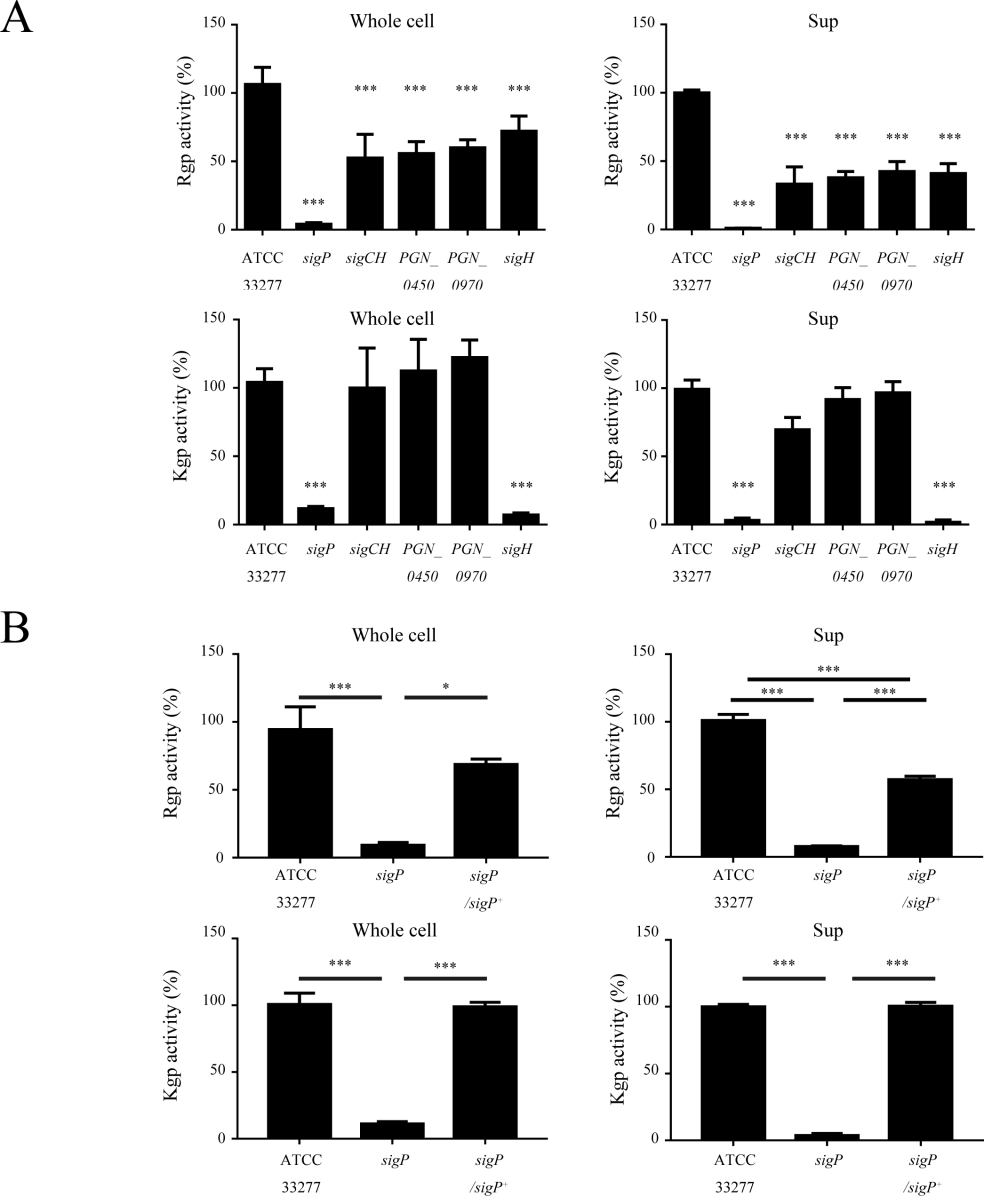
Gingipain activities of the indicated *Porphyromonas gingivalis* strains. Following 48 h of growth, *P*. *gingivalis* cultures were centrifuged. Gingipain activities in “culture supernatants” and “whole cells” of ECF sigma factor mutants (A) or the *sigP* mutant and its complemented strain (B) were normalized to those of *P*. *gingivalis* ATCC 33277, with the latter considered 100%. Data are expressed as means ± SD of nine independent experiments. *, *p* < 0.05; ***, *p* < 0.001.

A previous study suggested an association between HA and gingipain activity [34]. We therefore examined Rgp and Kgp activities in whole cells and culture supernatants of ECF sigma factor mutants. Rgp and Kgp activities were almost completely abolished in both whole cell and supernatant samples of the *sigP* mutant (Fig 3A). The Rgp activities of other ECF sigma factor mutants were approximately half (whole cells) or 40% (supernatants) of those of the wild type. In addition, Kgp activity was significantly reduced in both whole cells and supernatant of the *sigH* mutant. Complementation of the *sigP* mutant with wild-type *sigP* restored Kgp and partially Rgp activity (Fig 3B).

**Fig 4:**
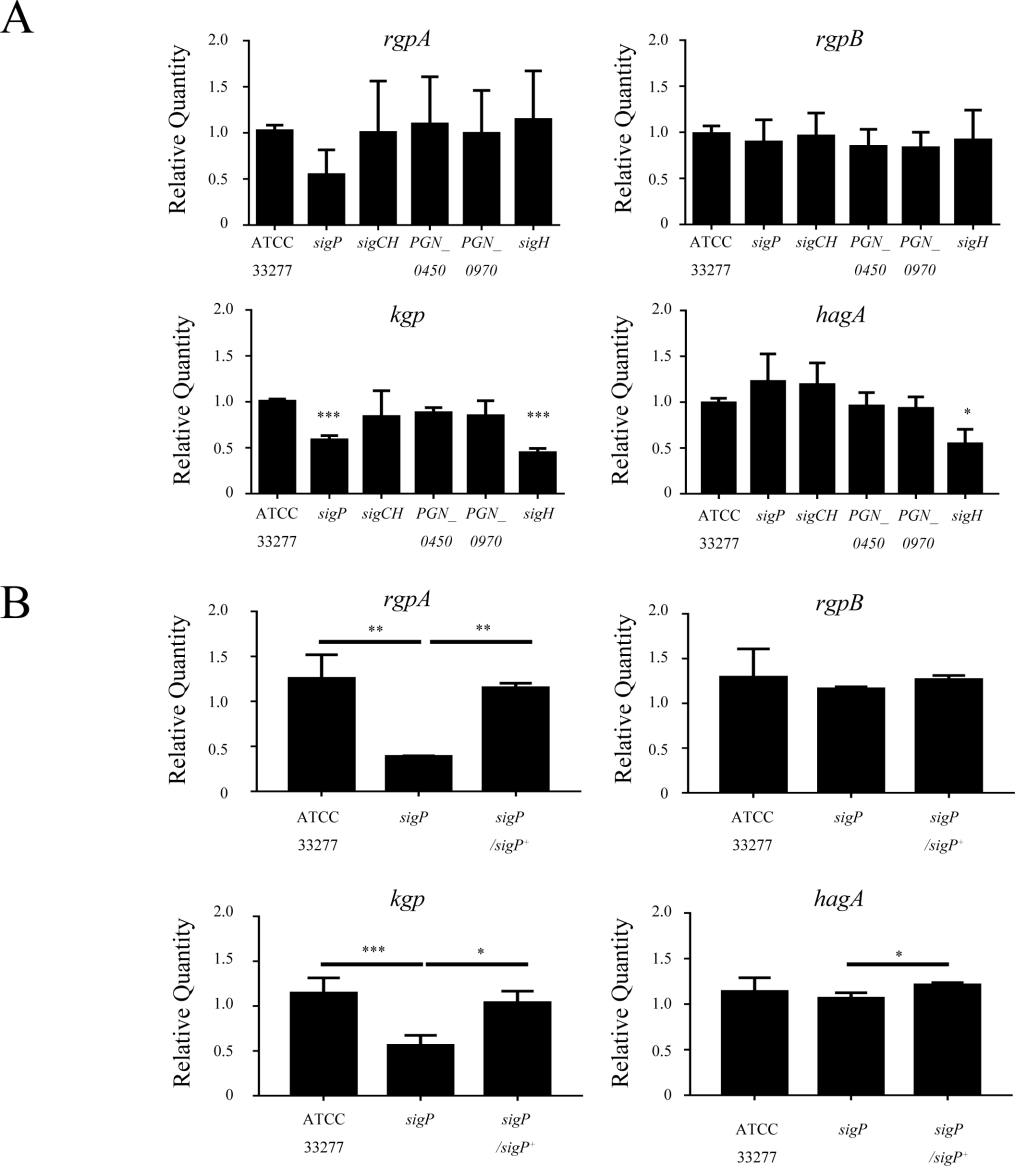
Expression levels of *rgpA*, *rgpB*, *kgp*, and *hagA* in the indicated *Porphyromonas gingivalis* strains. qRT-PCR analysis of the *rgpA*, *rgpB*, *kgp*, and *hagA* genes in ECF sigma factor mutants (A) or the *sigP* mutant and its complemented strain (B). Total RNA was extracted from *P*. *gingivalis* cells grown to an OD_660_ of 0.6–0.8. All assays were performed nine times, and means ± SD (standard deviations) are shown. *, *p* < 0.05; **, *p* < 0.01; ***, *p* < 0.001.

Two separate genes (*rgpA* and *rgpB*) and a single gene (*kgp*) have been shown to be involved in Rgp and Kgp activity [35]. In addition, the Hgp44 domain of RgpA, Kgp, and HagA mediate hemagglutination [33]. Therefore, *rgpA*, *rgpB*, *kgp*, and *hagA* were analyzed by qRT-PCR. The results showed that *kgp* was downregulated in the *sigP* and *sigH* mutants, whereas *rgpA* and *rgpB* exhibited almost the same levels of transcription as that of the wild type (Fig 4A). *hagA* expression was downregulated in the *sigH* mutant (Fig 4A). Complementation of the *sigP* mutant with wild-type *sigP* restored *kgp* expression (Fig 4B).

### ECF sigma factor deletions affect *P*. *gingivalis* MICs of ampicillin but not tetracycline or ofloxacin

Table 3 shows MIC values of the wild-type and ECF sigma factor mutant strains. Three antimicrobial agents were tested. All mutants showed the same levels of susceptibility to tetracycline and ofloxacin as those of the wild-type strain. Compared with the wild type, susceptibility to ampicillin was increased in the *sigP* mutant and decreased in the *PGN_0450* and *sigH* mutants. Susceptibility to ampicillin of the *sigP*-complemented strain was not different from that of the wild type.

**Table 3:** Antimicrobial susceptibilities of the *Porphyromonas gingivalis* ECF sigma factor mutants.

	MIC (μg/mL)		
Strain	Ampicillin	Tetracycline	Ofloxacin
*P*. *gingivalis* ATCC 33277	1.0	0.25	0.25
*P*. *gingivalis sigP* mutant	0.5	0.25	0.25
*P*. *gingivalis sigCH* mutant	1.0	0.25	0.25
*P*. *gingivalis PGN_0450* mutant	2.0	0.25	0.25
*P*. *gingivalis PGN_0970* mutant	1.0	0.25	0.25
*P*. *gingivalis sigH* mutant	> 2.0	0.25	0.25

### OMV formation and intermembrane distances are affected in some ECF sigma factor mutants

**Fig 5:**
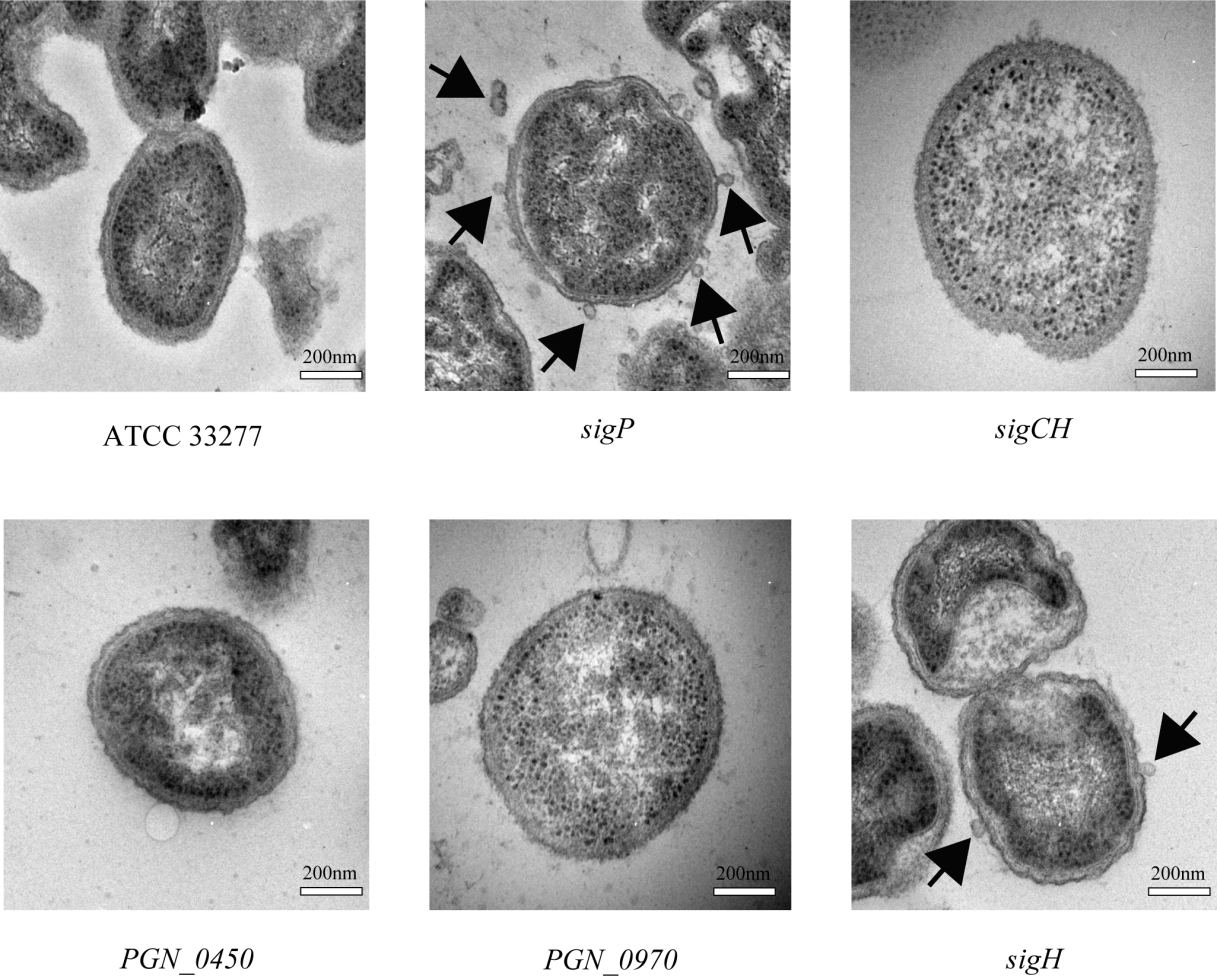
Transmission electron microscopy images of *Porphyromonas gingivalis* wild-type and ECF sigma factor mutant strains. The cells were stained for less than 1 min in 4% uranyl acetate and 0.2% lead citrate solutions. Scale bars are shown. The arrows indicate outer membrane vesicles.

**Fig 6:**
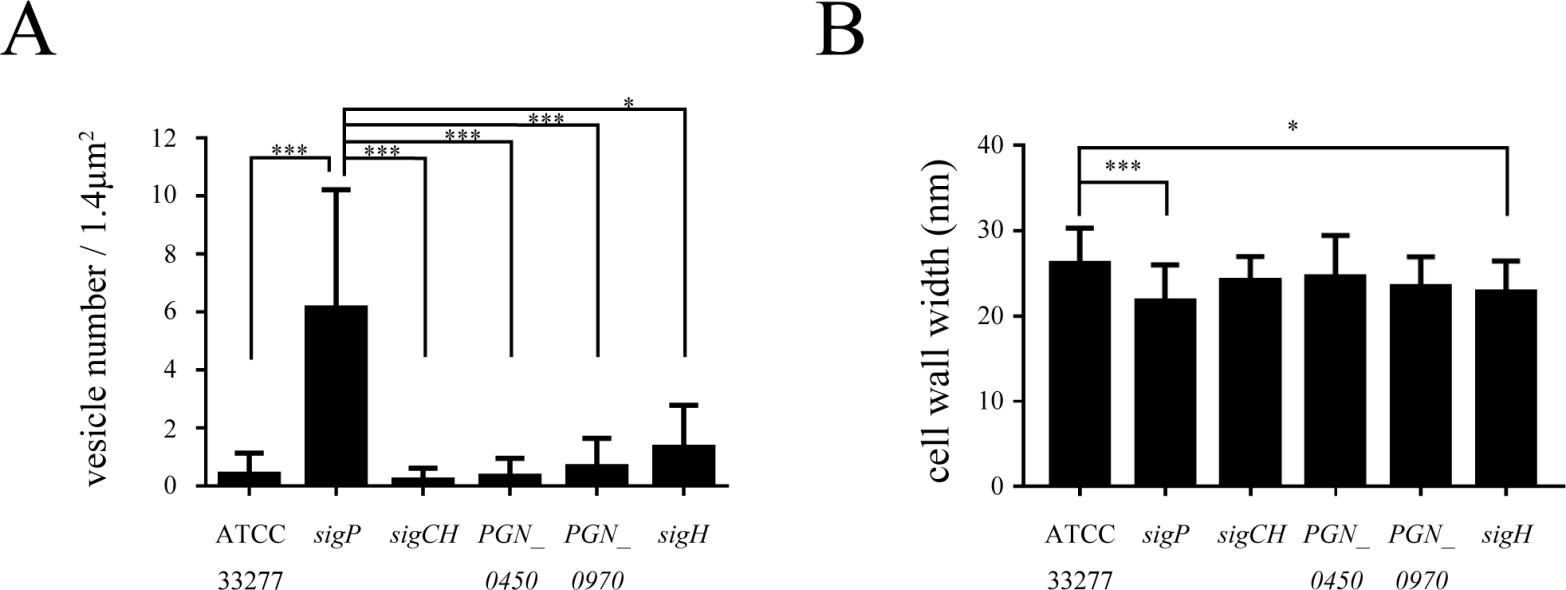
Vesicle numbers and intermembrane distances in *Porphyromonas gingivalis* wild-type and ECF sigma factor mutant strains. The number of vesicles per 1.4 μm^2^ (15 specimen counted) (A) and inter-membrane distance (distance between the outer and cytoplasmic membranes) (at least 20 specimen counted) (B) were established from TEM images. Data are expressed as means ± SD. *, *p* < 0.05; ***, *p* < 0.001.

TEM images of *P*. *gingivalis* wild type and mutant strains are shown in Fig 5. OMV formation at the cell surface was more pronounced in the *sigP* mutant than in the wild-type strain. The number of OMVs formed by the *sigP* mutant was significantly higher than that in the wild type (Fig 6A), and inter-membrane distances were significantly reduced in the *sigP* mutant (Fig 6B).

### Influence of SigP ECF sigma factor on the virulence of *P*. *gingivalis* W83

**Fig 7:**
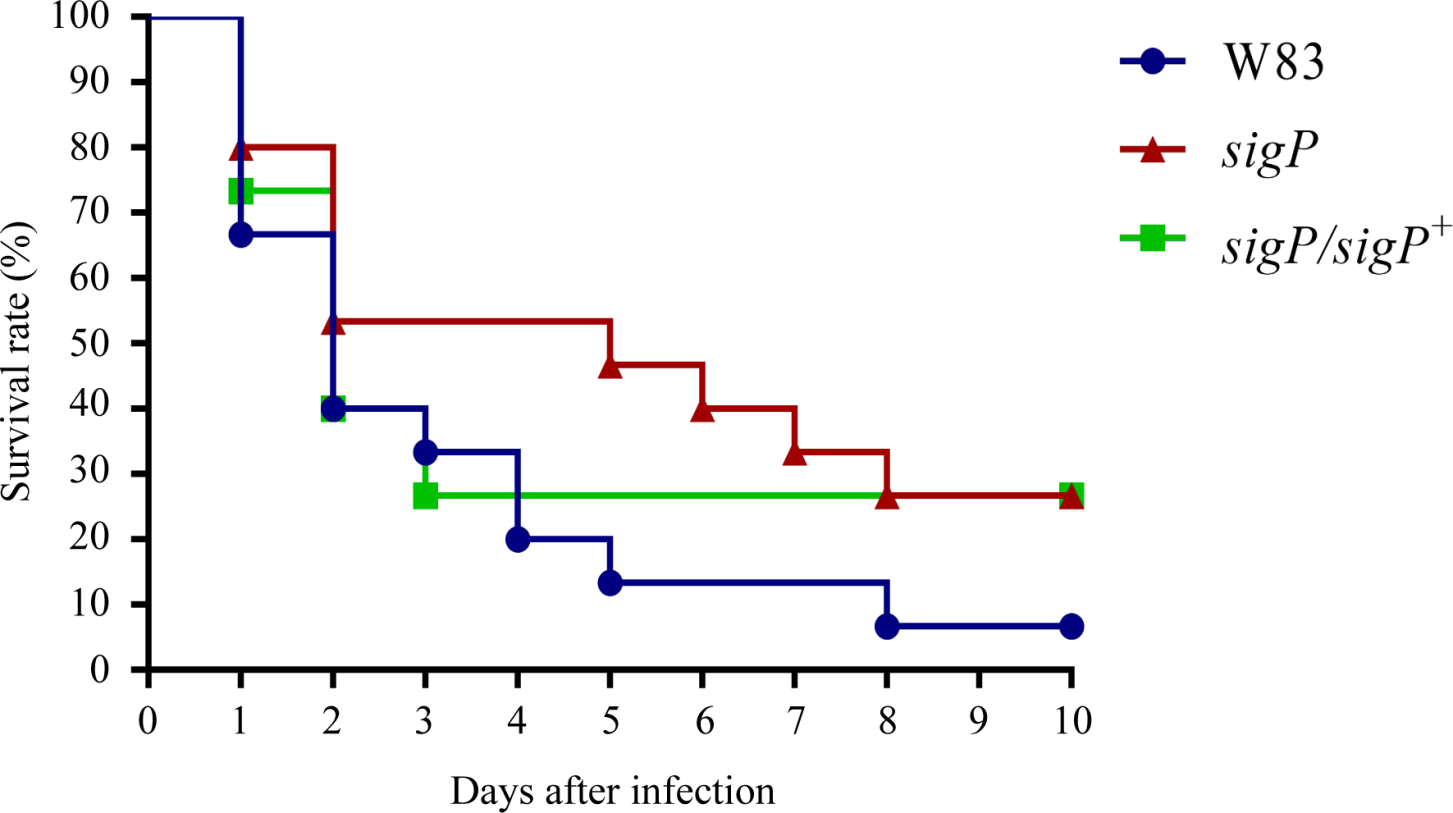
Virulence of *Porphyromonas gingivalis* W83 and the *sigP* mutant strain in a murine model. Five BALB/c mice were inoculated subcutaneously with 0.1 mL of bacterial suspension at two sites on the depilated dorsal surface (0.2 mL per mouse), and the survival of the mice was monitored daily for up to 10 days. Three sets of experiments were carried out (15 mice in total). For the data analysis, Kaplan-Meier plots were constructed, and the log rank test was used to evaluate the differences in mean survival rates between mice infected with the W83 parent strain, *sigP* mutant, and complemented strain in three experiments.

BALB/c mice were inoculated subcutaneously with 0.1 mL of bacterial suspension (2 × 10^10^ CFU per animal), and their survival was monitored for 10 days. The mouse mortality resulting from subcutaneous injection of *P*. *gingivalis* W83 and the *sigP* mutant and *sigP*-complemented strain is summarized in Fig 7. The log rank test showed no relationship between *P*. *gingivalis* virulence and the SigP ECF sigma factor.

## Discussion

In the present study, we determined that autoaggregation, hemagglutination, gingipain activities, and OMV formation, which all contribute to *P*. *gingivalis* virulence, were altered in the *sigP* mutant. Compared with the wild-type strain, Kgp activity was reduced in the *sigH* mutant, and Rgp activity was decreased in all mutants. In addition, the *sigP*, *PGN_0450*, and *sigH* mutants displayed altered ampicillin MICs. These results suggested that adaption to various environmental changes governed by ECF sigma factors plays a key role in the virulence of *P*. *gingivalis*.

In the present study, the activities of Kgp and Rgps were reduced most in the *sigP* strain compared to the other strains. In a previous report, inactivation of PG0162 and PG1660 (PGN_0274 and PGN_0450 in strain ATCC 33277) resulted in a 50% decrease in gingipain activity, and an RT-PCR analysis showed no changes in the expression of *rgpA*, *rgpB*, and *kgp* gingipain genes in these mutants [14]. Gingipains are exported by a type IX secretion system [36]. SigP and the two-component system PorXY regulates type IX secretion systems [18]. This suggests that the precursor forms of Kgp and Rgps may accumulate in the periplasmic space of the *sigP* mutant. In addition, we found that the expression of *rgpA* and *kgp* were decreased in the *sigP* mutant (Fig 4B). Kadowaki et al. showed, based on a transcriptional analysis by microarray or qRT-PCR, that *rgpA*, *rgpB*, and *kgp* did not change significantly in the *sigP* mutant [18]. Nevertheless, we found that the expression of *rgpA* and *kgp* decreased by approximately 60% in the *sigP* mutant and was restored in the complemented strain (Fig 4B). The exact reasons for this discrepancy in gene expression results are not known. However, we propose three possible reasons. First, microarray and qRT-PCR data often disagree [37, 38]. Second, the methods of *sigP* mutagenesis used were not the same. We used an erythromycin resistance cassette containing both *ermF* and *ermAM*, whereas Kadowaki et al. used only a *ermF* cassette. Third, Kadowaki et al. analyzed expression by qRT-PCR using SYBR Green dye, whereas we used a Taqman probe assay. However, we believe that our qRT-PCR data is reliable, because the complementing the *sigP* mutant with wild-type *sigP* restored *rgpA* and *kgp* expression. Further analysis is required to clarify whether SigP is involved in the regulation of *rgpA* and *kgp*. RgpA, Kgp, and HagA possess adhesion and hemagglutinin domains (Hgp44), and inactivation of *rgpA* significantly reduced HA [33, 39]. However, *hagA* expression was not downregulated in the *sigP* mutant. Thus, the reduction in HA may also be attributable to the decrease in gingipain activity of the *sigP* mutant.

Although the gingipain activities of *sigP* and *PGN_0450* mutants in the present study were different, those of *sigP* and *PG1660* mutants in the *P*. *gingivalis* W83 background were similar to what has been reported previously [14]. The difference between the present and previous studies may be attributable to the experimental conditions used. A difference was also observed in HA. Insertion Sequences (ISs) and miniature inverted-repeat transposable elements were detected in the *P*. *gingivalis* genome, and genome rearrangements were detected [12, 40]. Variations in fimbriae and capsules were reported [41, 42], as well as differences in the virulence of strains [43]. Further study is needed to clarify the relationship between ECF sigma factors and gingipain activity.

Autoaggregation of the *sigP* mutant was significantly reduced compared to that of the wild-type strain. Our results suggested that the *sigP* mutant was less hydrophobic than the wild type and the complemented strain (Fig 1D). Several studies have indicated that the hydrophobicity of bacterial cells is related to autoaggregation. However, autoaggregation of the *sigP*-complemented strain was not as same as that of the wild type (Fig 1C). Autoaggregation in *P*. *gingivalis* might be correlated with the bacterial surface hydrophobicity, fimbriae, capsule, VimA, Kgp activity [32, 44–47]. First we speculated that another factor, *P*. *gingivalis* FimA fimbriae, contributed little to autoaggregation. To investigate the relationship between autoaggregation and FimA, we performed FimA polymerization using SDS-polyacrylamide gel electrophoresis under nonreducing conditions. However, there was no obvious alteration of ladder-like bands in all of strains, suggesting that autoaggregation of the *sigP* mutant cannot be explained by its FimA polymerization (S1 Fig). Further analysis will be required to clarify the autoaggregation mechanism.

In a previous study, susceptibility to antimicrobial agents changed because of efflux pump inactivation [26]. We used the same antimicrobial agents to investigate the role of the ECF sigma factors in susceptibility to antimicrobials. The MIC of ampicillin was lower with the *sigP* mutant than with wild type. In contrast, the MICs of ampicillin with the *PGN_0450* and *sigH* mutants were higher. Ampicillin inhibits a transpeptidase involved in peptidoglycan synthesis. Intermembrane distances were also altered in *sigP* and *sigH* mutants. These results suggested that SigP and SigH affect cell wall structure in different ways. The expression of approximately 24% of *P*. *gingivalis* genes was affected by PG0162 overexpression [17]. It is possible that the three ECF sigma factors are involved in cell wall assembly. In *Staphylococcus aureus*, ECF sigma factor SigS was shown to be involved in the cellular response to cell wall-targeting antimicrobial agents [48]. Further research is needed to determine whether this mechanism plays a role in *P*. *gingivalis*.

The MICs of tetracycline and ofloxacin were the same for the wild type and all mutants tested. This indicated that the five ECF sigma factors were not involved in the response to stress caused by these antimicrobial agents. SigX, an ECF sigma factor in *Pseudomonas aeruginosa*, was reported to affect the efflux pump MexXY/OprM, a key element of bacterial adaptation to ribosome-targeting antibiotics [49, 50]. Inactivation of the *P*. *gingivalis* efflux pump was reported to increase susceptibility to ampicillin, tetracycline, and norfloxacin [51]. Our results, therefore, suggest that the ECF sigma factors examined are not involved in regulation of an efflux pump specific for removal of these antibacterial agents.

The cell wall of the *sigP* mutant was obviously different from that of the wild-type strain and was characterized by increased OMV formation. There are two possible explanations for the increase in OMVs. One reason is that disruption of σ^E^-regulated genes caused increased vesiculation in *Escherichia coli* [52]. Another reason is that overproduction of outer membranes relative to peptidoglycans caused blebbing of outer membranes [53]. Gui et al. [54] reviewed that the biogenesis of OMVs of *P*. *gingivalis* and another bacterium, *E*. *coli*. Overproduction of OMVs in *E*. *coli* was found to be related to ECF sigma factor (σ^E^) stress-response pathways. σ^E^ controls the expression of *degP*, which encodes a periplasmic serine protease. The DegP mutant accumulates misfolded proteins in the periplasm, increasing envelope stress, and, as a result, induces OMVs production. Therefore, we speculated that misfolded proteins or precursor forms of Kgp and Rgps may accumulate in the periplasmic space of the *sigP* mutant, causing increased budding of vesicles. In the present study, the ampicillin MIC of the *sigP* mutant was decreased relative to that of the wild type, suggesting that cell wall synthesis was affected by SigP inactivation. It is possible that the mutation affected the equilibrium between peptidoglycans and outer membrane components. The mechanism by which OMV formation increased in the *sigP* mutant should be investigated further.

As revealed by the mouse virulence assay, disruption of the *sigP* did not affect *P*. *gingivalis* virulence in mice. Previous studies have shown that Rgp and Kgp activity significantly reduced mouse virulence [30, 55]. This result may be attributed to an increase in a virulence factor other than Rgp and Kgp in the absence of SigP. However, we cannot yet explain why the virulence of the wild type and *sigP* mutant were similar in the mouse virulence assay. Further research is needed to clarify this finding.

Taken together, results of our study indicate that ECF sigma factor SigP is involved in regulating *P*. *gingivalis* virulence factors, including gingipain activity, autoaggregation, hemagglutination, and OMV formation.

## Supporting information

S1 Fig Polymerization patterns of FimA in the *sigP* mutant and the *sigP*-complemented strain. Whole-cell lysates were obtained from *P*. *gingivalis* ATCC 33277, *sigP* mutant and its complemented strain. Solubilized samples in the presence of β-mercaptoethanol were denatured at 100°C for 10 min, or 80°C for 10 min and then separated by SDS-PAGE and detected by anti-FimA antibody.
(TIF)
