# Supplementary figures and images for: Effect of extracytoplasmic function sigma factors on autoaggregation, hemagglutination, and cell surface properties of Porphyromonas gingivalis
Source: PLoS One. 2017 Sep 20;12(9):e0185027. doi: 10.1371/journal.pone.0185027 (PMC5607195; doi:10.1371/journal.pone.0185027)

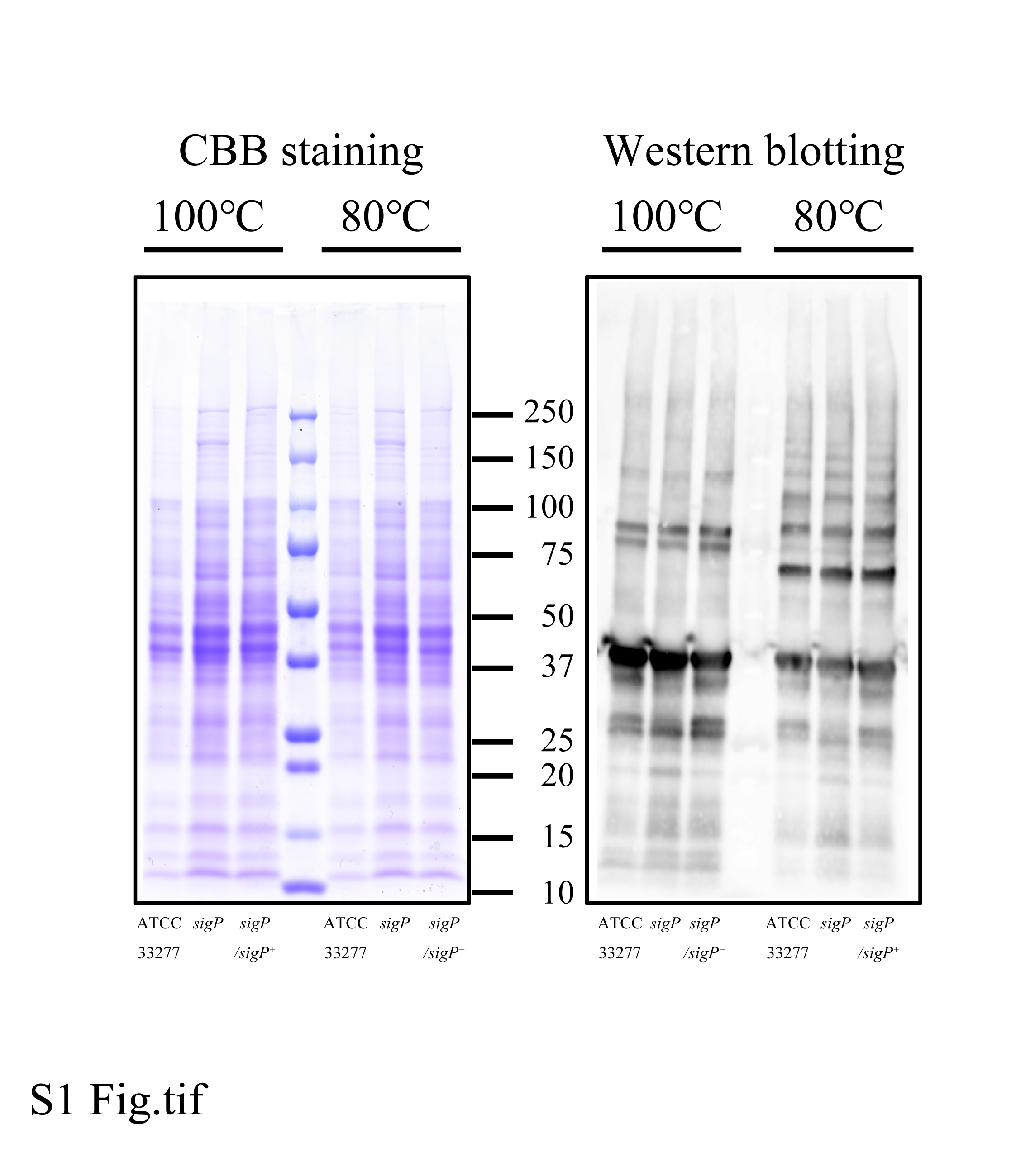

Supplement: S1 Fig — Whole-cell lysates were obtained from P. gingivalis ATCC 33277, sigP mutant and its complemented strain. Solubilized samples in the presence of β-mercaptoethanol were denatured at 100°C for 10 min, or 80°C for 10 min and then separated by SDS-PAGE and detected by anti-FimA antibody. (TIF) [file pone.0185027.s001.tif]
